# Clinical, laboratory and ultrasonographic findings differentiating low‐grade intestinal T‐cell lymphoma from lymphoplasmacytic enteritis in cats

**DOI:** 10.1111/jvim.16272

**Published:** 2021-10-23

**Authors:** Valérie Freiche, Julien Fages, Mathieu Victor Paulin, Julie Bruneau, Lucile Couronné, Alexander J. German, Dominique Penninck, Olivier Hermine

**Affiliations:** ^1^ Ecole Nationale Vétérinaire d'Alfort, CHUVA Unité de Médecine Interne Maisons‐Alfort France; ^2^ Centre DMV Lachine Quebec Canada; ^3^ Department of Small Animal Clinical Sciences Western College of Veterinary Medicine—University of Saskatchewan Saskatoon Saskatchewan Canada; ^4^ Pathology Department Hôpital Necker—Enfants Malades, Assistance Publique—Hôpitaux de Paris (APHP), Laboratory of Cellular and Molecular Mechanisms of Hematological Disorders and Therapeutical Implications, INSERM U1163, Imagine Institute, University of Paris Paris France; ^5^ Cytogenetics Department Hôpital Necker—Enfants Malades, Assistance Publique—Hôpitaux de Paris (APHP), Laboratory of Cellular and Molecular Mechanisms of Hematological Disorders and Therapeutical Implications, INSERM U1163, Imagine Institute, University of Paris Paris France; ^6^ Institute of Life Course and Medical Sciences University of Liverpool Merseyside United Kingdom; ^7^ Diagnostic Imaging Section, Department of Clinical Sciences Cummings School of Veterinary Medicine at Tufts University Grafton Massachusetts USA; ^8^ Hematology Department Hôpital Necker—Enfants Malades, Assistance Publique—Hôpitaux de Paris (APHP); Laboratory of Cellular and Molecular Mechanisms of Hematological Disorders and Therapeutical Implications, INSERM U1163, Imagine Institute, University of Paris Paris France

**Keywords:** alimentary lymphoma, cat, chronic enteropathy, full‐thickness intestinal biopsies, inflammatory bowel disease, ultrasonography

## Abstract

**Background:**

Low‐grade intestinal T‐cell lymphoma (LGITL) is the most common intestinal neoplasm in cats. Differentiating LGITL from lymphoplasmacytic enteritis (LPE) is challenging because clinical signs, laboratory results, diagnostic imaging findings, histology, immunohistochemistry, and clonality features may overlap.

**Objectives:**

To evaluate possible discriminatory clinical, laboratory and ultrasonographic features to differentiate LGITL from LPE.

**Animals:**

Twenty‐two cats diagnosed with LGITL and 22 cats with LPE based upon histology, immunohistochemistry, and lymphoid clonality.

**Methods:**

Prospective, cohort study. Cats presented with clinical signs consistent with LGITL or LPE were enrolled prospectively. All data contributing to the diagnostic evaluation was recorded.

**Results:**

A 3‐variable model (*P* < .001) consisting of male sex (*P* = .01), duration of clinical signs (*P* = .01), and polyphagia (*P* = .03) and a 2‐variable model (*P* < .001) including a rounded jejunal lymph node (*P* < .001) and ultrasonographic abdominal effusion (*P* = .04) were both helpful to differentiate LGITL from LPE.

**Conclusions and Clinical Importance:**

Most clinical signs and laboratory results are similar between cats diagnosed with LGITL and LPE. However, male sex, a longer duration of clinical signs and polyphagia might help clinicians distinguish LGITL from LPE. On ultrasonography, a rounded jejunal lymph node, and the presence of (albeit small volume) abdominal effusion tended to be more prevalent in cats with LGITL. However, a definitive diagnosis requires comprehensive histopathologic and phenotypic assessment.

**Abbreviations**
AL: alimentary lymphoma
GIL: gastrointestinal lymphoma
HGAL: high‐grade alimentary lymphoma
LGAL: low‐grade alimentary lymphoma
LGITL: low‐grade intestinal T‐cell lymphoma
LGLL: large granular lymphocytic lymphoma
LPE: lymphoplasmacytic enteritis

## 1 INTRODUCTION

Gastrointestinal lymphoma (GIL) is the most common digestive tract neoplasm in cats,^1^ and the most common anatomical form of lymphoma (50%‐75%) in this species.^1^, ^2^, ^3^, ^4^, ^5^ The current classification system for GIL originally was defined in 2012 and comprises 3 entities^5^, ^6^, ^7^: first, mucosal lymphoma which previously was named low‐grade alimentary lymphoma (LGAL) and recently renamed low‐grade intestinal T‐cell lymphoma (LGITL)^8^; second, transmural lymphoma, which is more frequently high‐grade alimentary lymphoma (HGAL) and characterized by parietal infiltration of small or large lymphocytes of B‐ or T‐cell type; and third, the least frequent but most aggressive form named large granular lymphocytic lymphoma (LGLL). Low‐grade intestinal T‐cell lymphoma is the most frequent subtype of GIL in cats, accounting for 60% to 75% of cases, and with increasing prevalence reported over the last 10 years.^7^ This disease is characterized by diffuse infiltration of neoplastic T‐lymphocytes, typically in the small intestine, with or without jejunal lymph node involvement.^5^, ^9^ The key clinical features of LGITL recently have been reviewed.^5^, ^7^, ^10^, ^11^, ^12^, ^13^ The condition is defined by persistent or recurrent gastrointestinal signs (weight loss, lethargy, vomiting, anorexia, diarrhea), progressing over months or years, and mainly affecting older cats (median age, 13 years) of any breed and sex.^5^, ^7^, ^10^, ^11^, ^12^, ^13^ Differentiating LGITL from lymphoplasmacytic enteritis (LPE) is challenging because clinical signs, laboratory results, diagnostic imaging findings, histology, immunohistochemistry and clonality features all overlap.^5^, ^7^, ^14^, ^15^, ^16^ Accurate diagnosis is made even more challenging by the finding that concurrent LPE has been described in up to 60% of alimentary lymphoma (AL) cases,^7^, ^17^ and it also has been suggested that LGITL might develop from LPE.^7^, ^12^, ^16^, ^18^, ^19^, ^20^, ^21^, ^22^, ^23^ Diagnostic investigations for AL include CBC, serum biochemistry, measurement of serum concentrations of B vitamins, diagnostic imaging, and intestinal biopsy. Given its widespread availability and noninvasive nature, abdominal ultrasonography is a common diagnostic imaging modality for cats presenting with gastrointestinal signs. Ultrasonographic findings previously reported for LGITL include a thickened gastrointestinal wall, altered wall layering and jejunal lymphadenopathy, although a thickened muscularis layer relative to the other layers is most common.^12^, ^24^, ^25^, ^26^ Muscularis layer hypertrophy also has been reported in LPE, LGITL and, less commonly, in mechanical obstruction.^27^ Despite available reference intervals for wall thickness in healthy cats,^28^, ^29^ such measurements cannot reliably distinguish LGITL from LPE. Jejunal lymph node size is also variable in cats with LGITL, and ultrasonographic features of jejunal lymphadenopathy have been associated with both LGITL and LPE.^26^ As a result, nodal ultrasonographic criteria have not yet discriminated LGITL from LPE.

Recently, new histologic and immunohistochemical features for LGITL have been suggested.^17^, ^30^ In a companion article,^31^ we reported new histopathologic criteria to discriminate LGITL from LPE in a cohort of cats. Using the same cohort, the current study aimed to determine whether discriminatory clinical, laboratory and ultrasonographic results can help differentiate LGITL from LPE.

## 2 MATERIALS AND METHODS

### 2.1 Study design and eligibility criteria

Our study was prospectively conducted at the same referral center, Alfort School of Veterinary Medicine, Paris, France, between July 2016 and July 2018.^31^ All data were prospectively acquired, except for ultrasonographic features, which were retrospectively and blindly reviewed. The study was approved by the ethical committee of our institution (ENVA COMERC n°2016‐05‐09), and all owners gave informed, written consent for all of the investigations. Cats were eligible for enrollment in the study if they displayed chronic (≥3 weeks) clinical signs suggesting a gastrointestinal disorder (vomiting, diarrhea, weight loss, lethargy, anorexia, or some combination of these), that did not improve after dietary modification and symptomatic treatment (parasiticides, gastrointestinal protectants, antibiotics). If a cat had been empirically treated with glucocorticoids before recruitment, a washout period of 3 weeks was mandatory before ultrasonographic examination and intestinal biopsy.

### 2.2 Acquisition of clinical and biological information

Standardized clinical data was gathered from all cats including age, sex, weight, nature and duration of clinical signs, diet history and abdominal palpation. Clinical data was collected by the same investigator (VF). Laboratory tests were performed on all cats including CBC, serum biochemistry panel, serum total thyroxine (T4) concentration, feline pancreas‐specific lipase (f‐PL), and serum cobalamin (vitamin B12) concentration. Blood samples were analyzed either by Idexx laboratories, France or BioPôle laboratory at Alfort School of Veterinary Medicine, Paris, France. A May‐Grunewald Giemsa‐stained blood smear was examined to confirm potential abnormalities, especially in cats with thrombocytopenia. The reference intervals for all variables from both laboratories are presented in Supplemental Table S1. Hypocobalaminemia was defined by serum cobalamin concentration <200 ng/L. Feline retroviral status was determined by rapid enzyme‐linked immunosorbent assays (SNAP Combo Plus FeLV Ag/FIV Ab test; Idexx Laboratories, Inc).

### 2.3 Abdominal ultrasonography

All representative ultrasonographic images were obtained with the same ultrasound machine (Affinity 50G, Philips, Amsterdam, Netherland), with both a 5‐8 MHz microconvex transducer and 5‐18 MHz linear array transducer. Standardized images were collected and ultrasonographic variables are presented in Table 1. A board‐certified radiologist (DP) and a senior radiology resident (JF) retrospectively reviewed all ultrasound images, without knowledge of the clinical history and final diagnosis. All measures used for statistical analysis (Table 1) were made at that point, with still ultrasonographic images available for review. The assessed anatomical regions of the gastrointestinal tract included stomach, duodenum, jejunum, ileum, and colon. For each location, total wall thickness was measured by placing calipers on the inner interface of the mucosa and on the outer aspect of the serosa. At each location, the maximal measurement as well as the thickness of the mucosa, submucosa and muscularis layers all were recorded. Relative wall thickness of each layer then was calculated as a ratio of the thickness of each respective layer relative to the maximal total wall thickness at that location. Finally, the ratio between muscularis thickness and submucosal thickness also was calculated. For the ileum, measurements from sagittal and transverse images were recorded separately and, for transverse images, distinct measurements were performed on the folds and in between the folds. For each gastrointestinal region, both loss of wall layering (defined as the complete absence of any wall layering) and alteration in wall layering (defined as any change in thickness or echogenicity of ≥1 layer) were evaluated and recorded as focal, multisegmental, or diffuse. Wall changes were considered focal when the affected segment was only visible in 1 scan view, multisegmental when >1 abnormal segments were identified, or diffuse when observed in all available images. Abdominal lymph nodes were evaluated for maximal thickness, echogenicity, and shape, including (when available) the jejunal, gastric, pancreaticoduodenal, and ileocolic lymph nodes.^32^ Maximal thickness was defined as the largest measurement perpendicular to the long axis of the lymph node, whereas echogenicity was assessed as normal, hypoechoic, or hyperechoic. Lymph node shape was subjectively recorded as normal, rounded, or irregular, whereas perinodal fat echogenicity was recorded as normal or hyperechoic. Abdominal effusion also was assessed and recorded as present or absent. When present, abdominal effusion was considered to be minimal when only visible as a scant volume in between a few small intestinal segments.

**TABLE 1:** Ultrasonographic data in cats with low‐grade intestinal T‐cell lymphoma (LGITL) or lymphoplasmacytic enteritis (LPE)

Variable	LGITL	LPE	*P* value^a^
Stomach			
Number	20	22	—
Total wall thickness (mm)	2.4 (1.5‐3.3)	2.3 (1.5‐4.3)	.21
Mucosal thickness (mm)	0.7 (0.4‐1.6)	0.7 (0.3‐1.4)	.63
(%)	33 (16‐57)	29 (15‐54)	.69
Submucosal thickness (mm)	0.6 (0.4‐1.9)	0.7 (0.3‐1.9)	.64
(%)	29 (14‐91)	29 (20‐91)	.6
Muscularis thickness (mm)	0.7 (0.2‐1.4)	0.6 (0.3‐0.9)	.29
(%)	29 (13‐44)	30 (13‐40)	.83
Altered wall layering	0/20	0/22	1
Duodenum			
Number	21	21	—
Total wall thickness (mm)	3.0 (2.2‐4.3)	2.9 (2.2‐3.6)	.68
Mucosal thickness (mm)	1.4 (0.8‐2.7)	1.2 (0.8‐2.0)	.76
(%)	44 (22‐74)	41 (28‐63)	.94
Submucosal thickness (mm)	0.7 (0.4‐1.1)	0.6 (0.3‐1.0)	.37
(%)	24 (12‐35)	23 (14‐33)	.62
Muscularis thickness (mm)	0.7 (0.2‐1.6)	0.7 (0.3‐1.4)	.97
(%)	22 (6‐46)	23 (12‐40)	.91
Altered wall layering	11/19 (58%)	9/21 (43%)	.34
Jejunum			
Number	22	22	—
Total wall thickness (mm)	3.2 (2.3‐4.9)	3.2 (2.1‐5.6)	.72
Mucosal thickness (mm)	1.4 (0.7‐2.3)	1.0 (0.4‐2.8)	.01
(%)	39.4 (2.9‐57.1)	33.2 (17.4‐60.9)	.02
Submucosal thickness (mm)	0.5 (0.3‐0.9)	0.6 (0.3‐1.4)	.72
(%)	16 (8‐27)	18 (11‐27)	.56
Muscularis thickness (mm)	1.1 (0.4‐2.4)	1.1 (0.5‐3.2)	.47
(%)	31 (17‐49)	37 (24‐61)	.08
Altered wall layering	21/22 (95%)	21/22 (95%)	1
Ileum			
Number	19	19	
Total wall thickness (mm)	3.3 (2.5‐5.7)	3.3 (2.2‐7.5)	.88
Mucosal thickness (mm)	1.3 (0.5‐1.8)	1.1 (0.5‐2.6)	.65
(%)	35 (19‐54)	30 (12‐48)	.45
Submucosal thickness (mm)	0.6 (.4‐1.7)	0.7 (0.4‐2.0)	.63
(%)	18 (12‐42)	22 (15‐69)	.44
Muscularis thickness (mm)	1.2 (0.7‐3.0)	1.4 (0.5‐2.9)	.53
(%)	33 (21‐53)	38 (17‐73)	.26
Altered wall layering	10/20 (50%)	11/17 (65%)	.23
Colon			
Number	21	20	
Total wall thickness (mm)	1.5 (0.9‐4.6)	1.3 (0.8‐3.9)	.08
Mucosal thickness (mm)	0.5 (0.2‐1.3)	0.4 (0.2‐1.3)	.11
(%)	31 (11‐50)	27 (17‐50)	.29
Submucosal thickness (mm)	0.4 (0.1‐2.3)	0.4 (0.2‐1.2)	.6
(%)	29 (16‐44)	29 (23‐50)	.36
Muscularis thickness (mm)	0.4 (0.1‐2.3)	0.3 (0.1‐1.4)	.25
(%)	27 (11‐50)	26 (13‐41)	.57
Altered wall layering	6/19 (32%)	3/18 (17%)	.45
Jejunal lymph nodes			
Number	20	17	
Thickness (mm)	6.7 (2.9‐12.0)	4.2 (1.8‐8.8)	.01
Rounded shape	17/20 (85%)	1/17 (6%)	<.001
Echogenicity^b^	13/20 (65%)	2/17 (12%)	<.001
Perinodal fat echogenicity^c^	14/20 (70%)	3/17 (18%)	.003
Ileocolic lymph nodes			
Number	15	12	—
Thickness (mm)	4.1 (1.4‐7.0)	2.4 (1.2‐5.5)	.05
Rounded shape	8/15 (53%)	5/12 (42%)	.55
Echogenicity^b^	7/15 (47%)	4/12 (33%)	.34
Perinodal fat echogenicity^c^	6/15 (40%)	2/12 (16%)	.4
Abdominal effusion	10/22 (45%)	3/22 (14%)	.02

*Note*: Continuous data are expressed as median (range) while categorical data are expressed as proportion (%).

^a^ Reported *P* values from continuous data are for the Mann‐Whitney test; reported *P* values for categorical data are either for χ^2^ test or Fisher's exact test (where expected values were <5 for at least 1 in the contingency table), with the level of statistical significance (bold red) set at *P* < .01 for 2‐sided analyses.

^b^ Indicates hypoechoic lymph node echogenicity.

^c^ Indicates hyperechoic perinodal fat echogenicity. LN: lymph node. The measurement in millimeters corresponds to the median of the group, with their corresponding minimal and maximal values in brackets. The value expressed in % corresponds to the aforementioned thickness divided by the total wall thickness and multiplied by 100.

### 2.4 Exclusion criteria

Cats were excluded from the study if another tumor was suspected before surgery or if other comorbidities were present that could lead to complications. Metabolic disorders were treated before enrollment in the study.

### 2.5 Collection of gastrointestinal biopsy material

Full‐thickness intestinal biopsy samples were collected at celiotomy in all but 3 cats the owners of which declined surgery; in those cats, biopsy samples instead were collected during endoscopy. Samples were taken from the duodenum, jejunum, ileum, or a combination of these intestinal segments. Biopsy sites were chosen according to sonographic features (eg, parietal thickening, especially when the ratio of muscularis‐to‐submucosa thickness was >1)^24^ in addition to visual and tactile inspection of intestinal loops during celiotomy (induration and color change). Jejunal lymph node biopsy samples also were collected if nodes were enlarged on ultrasonographic assessment. In the cats in which endoscopy was used, a combined upper and lower endoscopic procedure was performed, and 6 to 8 biopsy samples per intestinal segment were collected.^33^ Cats were hospitalized for 48 to 96 hours after the procedure to monitor for postoperative complications. All cats underwent serial measurements of hematocrit and serum albumin concentration after surgery, and abdominal ultrasonography was repeated before discharge in all cases to detect any surgical complications.

### 2.6 Histopathology

Intestinal biopsy samples were fixed in neutral‐buffered formalin and submitted to a human medical hospital pathology department (Hôpital Necker‐Enfants Malades, Hôpitaux de Paris, University of Paris, France) and a veterinary pathology department (Alfort School of Veterinary Medicine, BioPôle, Paris, France) for comparative histologic evaluation. Specimens were processed routinely, embedded in paraffin, and cut into 4 μm‐thick sections. Tissue sections stained with hematoxylin & eosin (HE) and Masson's trichrome were blindly reviewed by a board‐certified veterinary anatomic pathologist (NC) and a human medical anatomic pathologist researcher (JB), who also was an expert in intestinal lymphoproliferative disorders of humans. The epithelium and lamina propria were reviewed separately, as presented in the companion article.^31^ Because human medical and veterinary classification systems tend to use overlapping criteria, samples were classified according to criteria of the World Small Animal Veterinary Association (WSAVA) Gastrointestinal Standardization Group,^34^ the revised 2016 World Health Organization (WHO) classification of lymphoma in humans,^34^ the WHO classification of lymphoma in dogs and using a veterinary textbook.^35^ As presented in the companion article,^31^ clonality results were discussed with a human medical specialist in lymphoproliferative disorders (Maria‐Elena Turba, Laboratorio Genefast, Forli, Italy). Uncertain cases were characterized by criteria applicable to both diseases (eg, coexistence of lymphocytic and neutrophilic cryptitis, monomorphic small lymphocytes within a polymorphic background). These cases ultimately were discussed between both pathologists to find an agreement, as discussed in the companion article.^31^

### 2.7 Data analysis and statistics

All statistics were performed by using computer softwares (JMP version 14.3.0, SAS Institute, Inc, Cary, North Carolina; GraphPad PRISM version 8.1.1, San Diego, California). Continuous variables (eg, maximal total wall thickness, relative wall thickness, lymph nodes thickness) were visually and qualitatively assessed for normality by the Shapiro‐Wilk test. In all cases, the assumption of equal variances was tested and not rejected. Where data were normally distributed, group comparisons (ie, LPE vs LGITL) were tested by means of a 2‐sample independent *t* test assuming equal variances, whereas the Mann‐Whitney test was used where data were not normally distributed. For categorical variables, 2‐by‐2 contingency tables were constructed, and groups were compared by χ^2^ test or Fisher's exact tests when expected counts within cells were <5. For such analyses, ordinal categorical variables were converted into binary variables where normal was assigned a score of 0 and abnormal results a score of 1. Multiple nominal logistic regression was used to explore associations between disease group and a range of clinical and ultrasonographic variables. Given the number of variables studied, separate multiple regression models initially were built for clinical and ultrasonographic variables, each of which initially included variables that were significant at *P* < .05 in simple logistic regression. These *P* values were often different from those obtained with the other statistical methods used in initial analyses (ie, Mann‐Whitney tests, χ^2^ test, and Fisher's exact tests). Competing models then were tested in a backwards and forwards stepwise fashion, interactions among variables were evaluated by contingency tables, and the best fit model chosen by the Bayesian Information Criterion (BIC). With this approach, variables are removal or added sequentially until the best fit model is found (ie, the model with the smallest BIC), with evidence of superiority of 1 model over another assumed when the numerical difference in BIC between models was >2.^36^ Once separate logistic regression models had been constructed for clinical and ultrasonographic variables, a final model then was built combining both clinical and ultrasonographic variables. The initial model included all variables included in the final clinical and ultrasonographic models, with the model again refined by the same method for inclusion or rejection of variables until a best fit model was identified. For multiple regression analyses, the level of statistical significance selected was set at *P* < .05 for 2‐sided analyses.

## 3 RESULTS

### 3.1 Study cats

Twenty‐two cats were diagnosed with LGITL and 22 cats with LPE after histology, immunohistochemistry and clonality assessment, with full details reported in the companion article.^31^ Signalment data of cats diagnosed with LGITL or LPE are presented in Table 2. Most cats were domestic shorthair (LGITL: 18/22, 82%; LPE: 17/22, 77%; *P* > .99), with various other breeds also represented. In the LGITL group, there were 16/22 (73%) male and 6/22 (27%) female cats whereas, in the LPE group, there were 8/22 (36%) male and 14/22 (64%) female cats (*P* = .02). All cats were neutered. In the LGITL and LPE groups, median age was 13 years (range, 8‐16 years) and 11.5 years (range, 7‐15 years), respectively, and median weight was 4.0 kg (range, 2.2‐7.4 kg) and 3.7 kg (range, 2.1‐6.3 kg), respectively (*P* = .9). Full‐thickness intestinal biopsy samples were obtained from 21/22 LGITL and 20/22 LPE cats, with endoscopic biopsy samples being collected in the remaining 3 cats.

**TABLE 2:** Signalment and epidemiological data of cats diagnosed with low‐grade intestinal T‐cell lymphoma (LGITL) or lymphoplasmacytic enteritis (LPE)

Variable	LGITL	LPE	*P* value^a^
Number	22	22	
Breed	18 Domestic Shorthair, 2 Siamese, 1 Angora, 1 Norwegian Forest	17 Domestic Shorthair, 2 Persian, 1 Siamese, 1 Oriental, 1 Burmese	>.99
Male sex	16 (73%)	6 (27%)	.02
Neutered cats	22 (100%)	22 (100%)	>.99
Age (years old)	13 (8‐16)	11.5 (7‐15)	.34
Weight (kg)	4.0 (2.2‐7.4)	3.7 (2.1‐6.3)	.9
Exclusive indoor life	10 (45%)	9 (41%)	.76
Commercial diet	22 (100%)	22 (100%)	>.99

*Note*: Continuous data are expressed as median (range); categorical data are expressed as number (%).

^a^ Reported *P* values are for the Mann‐Whitney test (continuous data) or either χ^2^ or Fisher's exact test (proportional data).

### 3.2 Clinical data

Clinical data of cats diagnosed with LGITL or LPE are presented in Table 3. Clinical signs were present for longer in LGITL cats (median, 365 days; range, 62‐1460 days) compared with LPE cats (median, 107 days; range, 7‐1095 days; *P* < .001). Diarrhea of suspected small intestinal origin was reported in 14/22 (64%) cats with LGITL and in 6/22 (27%) cats with LPE (*P* = .02). No other significant differences between groups were identified by simple statistical analyses (Mann‐Whitney tests, χ^2^ tests, and Fisher's exact tests).

**TABLE 3:** Clinical data of cats diagnosed with low‐grade intestinal T‐cell lymphoma (LGITL) or lymphoplasmacytic enteritis (LPE)

Variable	LGITL	LPE	*P* value^a^
Weight loss	17/22 (77%)	13/22 (56%)	.2
Vomiting	15/22 (68%)	18/22 (82%)	.3
Alimentary vomiting	12/15 (80%)	11/19 (58%)	.27
Nonalimentary vomiting	12/15 (80%)	13/19 (68%)	.7
Small intestinal diarrhea	14/22 (64%)	6/22 (27%)	.02
Large intestinal diarrhea	4/22 (18%)	6/22 (27%)	.72
Hyporexia	10/22 (45%)	15/22 (68%)	.13
Lethargy	8/22 (36%)	13/22 (59%)	.13
Polyphagia	6/22 (27%)	1/22 (5%)	.1
Constipation	1/22 (5%)	2/22 (9%)	>.99
Hematochezia	1/22 (5%)	6/22 (27%)	.1
Melena	1/22 (5%)	1/22 (5%)	1
Duration of clinical signs (days)	365 (62‐1460)	107 (7‐1095)	<.001
Hyperthermia	1/22 (5%)	2/22 (9%)	>.99
Abnormal abdominal palpation	16/22 (73%)	18/22 (82%)	.72
Thickened intestinal loops	7/16 (44%)	12/18 (67%)	.18
Abdominal pain	4/16 (25%)	6/18 (33%)	.72

*Note*: Except for duration of clinical signs (median [range]), all data are expressed as proportion (%).

^a^ Reported *P* values are for either χ^2^ test or Fisher's exact test (where expected values were <5 for at least 1 in the contingency table).

### 3.3 Serum biochemistry

Serum biochemical variables are presented in Table 4. The number of cats with hypocobalaminemia (defined as a serum cobalamin concentration <200 ng/L, the lower limit of the reference interval) was 12/21 (57%) and 4/21 (19%) for LGITL and LPE cats, respectively (*P* = .01). No other significant differences between groups were identified by simple statistical analyses.

**TABLE 4:** Serum biochemical data in cats with low‐grade intestinal T‐cell lymphoma (LGITL) or lymphoplasmacytic enteritis (LPE)

Variable	LGITL	LPE	*P* value^a^
Albuminemia (g/dL)	3.0 (2.0‐5.0)	3.1 (2.2‐4.0)	—
Hypoalbuminemia	3/22 (14%)	7/22 (32%)	.28
Total proteinemia (g/dL)	7.3 (5.9‐10.7)	7.3 (5.2‐8.5)	—
Total hypoproteinemia	0/22 (0%)	3/22 (14%)	.23
ALT (U/L)	68 (22‐451)	50 (24‐100)	—
ALP (U/L)	41 (10‐262)	41 (10‐155)	—
Increased liver enzyme activity	3/22 (14%)	0/22 (0%)	.23
f‐PL (μg/L)	2.9 (1.11‐26)^b^	2.6 (0.5‐23)^c^	—
f‐PL > 3.5 (μg/L)	6/19 (32%)	8/22 (36%)	.75
Hypophosphatemia	2/21 (10%)	0/18 (0%)	.49
Hypocobalaminemia^d^	12/21 (57%)	4/21 (19%)	.01

*Note*: Continuous data are expressed as median (range); categorical data are expressed as proportion (%).

^a^ Reported *P* values are for either χ^2^ or Fisher's exact test (when expected counts within cells were <5).

^b^ Of 19 LGITL cats with available data.

^c^ Of 22 LPE cats with available data.

^d^ Defined as a cobalamin concentration <200 ng/L.

### 3.4 Hematology

Hematological data of cats diagnosed with LGITL or LPE are presented in Table 5. No significant differences between groups were identified by simple statistical analyses.

**TABLE 5:** Hematological data in cats with low‐grade intestinal T‐cell lymphoma (LGITL) or lymphoplasmacytic enteritis (LPE)

Variable	LGITL	LPE	*P* value^a^
Hemoglobinemia (mmol/L)	7.60 (4.65‐9.49)	7.14 (3.48‐9.62)	—
Anemia	4/22 (18%)	6/22 (27%)	.72
Leucocytes (G/L)	13.32 (5.69‐35.20)	11.47 (4.60‐38.02)	—
Leukocytosis	6/22 (27%)	4/22 (18%)	.72
Leucopenia	0/22 (0%)	0/22 (0%)	>.99
Neutrophils (G/L)	9.39 (2.38‐33.47)	7.58 (2.70‐29.43)	—
Neutrophilia	8/22 (36%)	5/22 (23%)	.32
Neutropenia	0/22 (0%)	1/22 (5%)	>.99
Eosinophils (G/L)	0.44 (0‐7.29)	0.62 (0‐3.94)	—
Eosinophilia	2/22 (9%)	3/22 (14%)	>.99
Eosinopenia	2/22 (9%)	2/22 (9%)	>.99
Lymphocytes (G/L)	1.63 (0.35‐10.22)	2.32 (0.38‐11.10)	—
Lymphocytosis	1/22 (5%)	1/22 (5%)	>.99
Lymphopenia	8/22 (36%)	5/22 (23%)	.32
Monocytes (G/L)	0.52 (0.18‐1.83)	0.36 (0.00‐1.84)	—
Monocytosis	9/22 (41%)	5/22 (23%)	.2
Monocytopenia	0/22 (0%)	1/22 (5%)	>.99

*Note*: Continuous data are expressed as median (range); categorical data are expressed as proportion (%).

^a^ Reported *P* values are for either χ^2^ or Fisher's exact test (when expected counts within cells were <5).

### 3.5 Virology

Only 1 cat (in the LGITL group) tested positive for FIV antibodies, and none of the cats tested positive for FeLV antigen.

### 3.6 Ultrasonography

**FIGURE 1:**
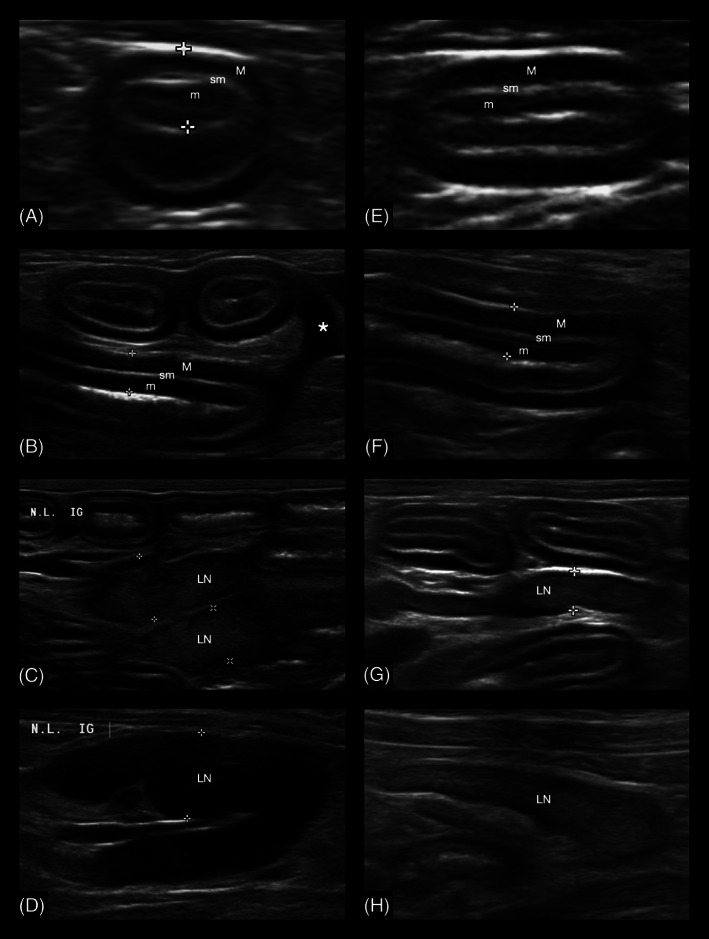
Examples of key ultrasonographic key features in cats with low‐grade intestinal T‐cell lymphoma (LGITL) or lymphoplasmacytic enteritis (LPE). The left (A‐D) column shows ultrasonographic images of 4 different cats diagnosed with LGITL. LN: lymph node; m: mucosa; M: muscularis; sm: submucosa. (A) Transverse image of a jejunal segment. Total wall thickness of this segment was 3.3 mm (distance between the white crosses), with a 1.8 mm thick mucosa and a 0.8 mm thick muscularis layer. (B) These jejunal segments have thickening of mucosal and muscularis layers. Note the presence of adjacent mild anechoic peritoneal effusion (*). (C) These 2 jejunal lymph nodes are mildly enlarged with a normal echogenicity. (D) This jejunal lymph node is mildly enlarged, measuring up to 7.9 mm in thickness (white crosses). It is also hypoechoic and has rounded margins. (E) A transverse image of a jejunal segment. The total wall thickness of this segment is 3.2 mm, with a 1.0 mm thick mucosa and a 1.3 mm thick muscularis layer. (F) Sagittal image of a jejunal segment exhibiting muscularis layer thickening. (G) This jejunal lymph node is normal in size, measures up to 4.2 mm in thickness (distance between white crosses), and also has both a normal shape and echogenicity. (H) This jejunal lymph node is normal in size, measuring up to 4.7 mm in thickness, and has a normal echogenicity. The margins of its caudal pole (on the right part of the image) are slightly rounded

Ultrasonographic data of cats diagnosed with LGITL and LPE are presented in Table 1, with examples of key ultrasonographic features in both LGITL and LPE cats shown in Figure 1. The retrospective review of ultrasonographic images did not always allow measurement of all evaluated structures on an individual basis. Therefore, Table 1 details for all assessed variables the total number for cats for which measurements were made.

#### 3.6.1 Gastrointestinal tract assessment

Total jejunal mucosal thickness was higher in LGITL (median, 1.4 mm; range, 0.7‐2.3 mm) than in LPE cats (median, 1.0 mm; range, 0.4‐2.8 mm; *P* = .009), but relative wall thickness was not (median, 39%; range, 29%‐57% in LGITL cats; median, 33%; range, 17%‐61% in LPE cats; *P* = .02). Although altered jejunal wall layering was common (LGITL: 21/22, 95%; LPE: 21/22, 95%; *P* = 1), loss of layering was rare (LGITL: 2/22, 2.9%; LPE: 0/22, 0%; *P* = .49). Furthermore, no differences were noted in any ultrasonographic variables from other regions of the gastrointestinal tract, including the stomach, duodenum, ileum, and colon (Table 1).

#### 3.6.2 Jejunal lymph nodes

Jejunal lymph nodes of LGITL cats were thicker (median, 6.7 mm; range, 2.9‐12.0 mm in LGITL cats; median, 4.2 mm; range, 1.8‐8.8 mm in LPE cats; *P* = .01), more frequently rounded (in 17/20 [85%] LGITL cats; in 1/17 [6%] LPE cats; *P* < .001), hypoechoic (in 13/20 [65%] LGITL cats; in 2/17 [12%] LPE cats; *P* < .001) and surrounded with hyperechoic perinodal fat (in 14/20 [70%] LGITL cats; in 3/17 [18%] LPE cats; *P* = .003), compared to those of LPE cats. For many other lymph node variables, either no group differences were seen or too few measurements were taken to enable meaningful statistical comparisons to be made.

#### 3.6.3 Abdominal effusion

Abdominal effusion was seen in 10/22 (45%) LGITL cats and in 3/22 (14%) LPE cats (*P* = .02).

### 3.7 Multiple logistic regression

#### 3.7.1 Clinical variables model

Initially, a multiple logistic regression model was built with the following 7 variables that were significant (at *P* < .05) on simple logistic regression: sex (*P* = .02), duration of clinical signs (*P* = .03), small intestinal diarrhea (*P* = .02), hematochezia (*P* = .03), polyphagia (*P* = .03), increased liver enzyme activity (*P* = .03), and hypocobalaminemia (*P* = .001). Using backwards and forwards stepwise regression, the best fit model (R^2^, 0.65; BIC, 40.2; *P* < .001) contained 4 variables: duration of clinical signs (*P* < .001), hematochezia (*P* < .001), sex (*P* < .001), and polyphagia (*P* = .02). However, examination of contingency tables indicated confounding between hematochezia and polyphagia, whereby none of the 7 cats with hematochezia had polyphagia and vice versa. As a result, estimates of odds ratios (OR) from this multiple regression were deemed to be unreliable (ie, inconsistent with OR from simple regression and some extreme values). The influence of confounding was resolved by removing hematochezia, resulting in a 3‐variable model (R^2^, 0.36; BIC, 53.8; *P* < .001) comprising duration of clinical signs (OR, 1.004; 95% confidence interval [CI], 1.001‐1.007; *P* = .01), sex (male vs female; OR, 20.0; 95% CI, 2.0‐159.7; *P* = .01), and polyphagia (OR, 28.9; 95% CI, 1.4‐576.9; *P* = .03).

#### 3.7.2 Ultrasonographic variables model

Initially, a multiple logistic regression model was built with the following 8 variables that were significant (at *P* < .05) on simple logistic regression: jejunal mucosa thickness (*P* = .03); jejunal lymph node thickness (*P* < .001); echogenicity (*P* < .001); shape (*P* < .001) and peripancreatic fat (*P* = .001); pancreaticoduodenal lymph node thickness (*P* = .003) and shape (*P* = .02); and abdominal effusion (*P* = .02). After refinement using backwards and forwards stepwise regression, 2 best‐fit models were found, which had a similar level of fit. The first contained 2 variables: jejunal lymph node shape (OR, 43.3; 95% CI, 4.4‐423.9; *P* < .001) and ultrasonographic abdominal effusion (OR, 8.8; 95% CI, 0.8‐95.6; *P* = .04), and was a marginally better fit (R^2^, 0.44; BIC, 39.3; *P* < .001) than the second (R^2^, 0.36; BIC, 39.9; *P* < .001), which only contained jejunal lymph node shape (OR, 29.2; 95% CI, 4.7‐183.4; *P* < .001).

#### 3.7.3 Combined clinical and ultrasonographic variables model

Initially, a multiple logistic regression model was built with 6 variables that were included in the separate regression models of clinical and ultrasonographic variables: duration of clinical signs, hematochezia, sex, polyphagia, jejunal lymph node shape and abdominal effusion. After refinement using backwards and forwards stepwise regression, 2 best‐fit models were found, which were the same as those created when including only ultrasonographic variables. In this respect, the first combined jejunal lymph node shape (OR, 43.3; 95% CI, 4.4‐423.9; *P* < .001) with ultrasonographic evidence of abdominal effusion (OR, 8.8; 95% CI, 0.8‐95.6; *P* = .04), and fitted marginally better (R^2^, 0.44; BIC, 39.3; *P* < .001) than the second (R^2^, 0.36; BIC, 39.9; *P* < .001), which only contained jejunal lymph node shape (OR, 29.2; 95% CI, 4.7‐183.4; *P* < .001).

## 4 DISCUSSION

Our prospective study compared the clinical, laboratory and ultrasonographic features of cats diagnosed with either LGITL or LPE. Although accurate diagnosis using gastrointestinal biopsy samples can be challenging, a strength of our study was the fact that diagnosis was confirmed by both histologic and immunohistochemical analyses blindly conducted in parallel by both a board‐certified veterinary pathologist and a specialized pathologist in human medicine.^31^ Such an approach should help minimize the chances that errors were made in group classification, thereby improving reliability of the results.^31^

Considering signalment data, more LGITL cats were male than in the LPE group. A male predisposition has been reported previously in cats with AL (all AL subtypes merged), although no association has been observed previously in the low‐grade subtype (LGITL).^3^, ^21^, ^37^, ^38^ Given such a discrepancy in findings, additional studies are required, including additional genomics studies. In contrast to sex, no group differences were seen for breed, where most cats were domestic shorthair as seen in a previous study,^15^ and no genetic predisposition has been reported previously.^7^ Also, no difference in age was found between groups. Although some authors suggest that AL predominantly affects older cats,^3^ whereas LPE affects cats of all ages,^11^, ^23^, ^38^, ^39^ these previous studies included all types of AL and not exclusively the LGITL subtype. Given that all cats in our study were >7 years old, which is typical of cats referred to our institution, selection bias should be considered. No difference in body weight was found between cats with LGITL and LPE, which perhaps is not surprising because weight loss was seen in both groups, and is a sign reportedly seen in most cats with LGITL or LPE.^3^, ^5^, ^11^, ^12^, ^40^

The median duration of clinical signs at the time of first consultation was longer in LGITL cats than in cats with LPE, which might be associated with difficulties faced by primary care veterinarians in diagnosing LGITL. Alternatively, a continuum might exist between LPE and LGITL pathogenesis, whereby some LPE cases progress to LGITL over time.^12^, ^21^, ^22^, ^41^ A recent publication proposed a new model of lymphomagenesis in indolent LGITL.^31^ As suggested by the histologic data, validating an apical‐to‐basal gradient, the initiating event would be chronic stimulation by a food antigen or an antigen from a bacterial or viral pathogen. An apical‐to‐basal gradient is characterized by a more severe apical than basal neoplastic cell infiltration within the intestinal mucosa.^31^ Such a continuum between LPE and LGITL could explain the difficulty in differentiating these 2 entities and would be similar to the situation in human patients, where some celiac diseases are suggested to have low‐grade epitheliotropic lymphoma rather than inflammatory proliferation.^42^, ^43^ Polyphagia was more common in cats with LGITL, whereas hematochezia was more common in cats with LPE. The latter might reflect the fact that large intestinal involvement is more likely in cats with LPE than in cats with LGITL, but the reason why polyphagia was more common in LGITL is unclear. No other differences were found in the presence of any other signs including lethargy, weight loss, anorexia, vomiting, large intestinal diarrhea, constipation, melena, and hyperthermia. These findings were consistent with those in the veterinary literature, where none of these signs is pathognomonic for either LGITL or LPE and these signs can be shared by many other conditions.^2^, ^4^, ^5^, ^7^, ^12^, ^13^, ^15^, ^16^, ^21^, ^22^, ^25^, ^44^, ^45^

Similar to clinical signs, few group differences were identified in physical examination findings. For example, although it was common for abnormalities to be identified on abdominal palpation (eg, thickened bowel loops, discomfort, or pain), no group differences were found. Some authors have suggested that abdominal palpation might be useful for high‐grade subtypes of AL (eg, HGAL and LGLL), where intestinal masses or severe lymphadenomegaly can be present.^4^, ^5^, ^45^

Mild hypoalbuminemia was found in 3 (14%) LGITL and 7 (32%) LPE cats, which is less common than reported in some other previous studies where hypoalbuminemia was present in 49%^46^ and 77%^46^ of LGITL and LPE cats, respectively. The reason for the differences between our study and previous studies is not known. Hypocobalaminemia was significantly more frequent in the LGITL group than in the LPE group. Besides these findings, no group differences for liver enzyme activity and fPLI concentration were found, suggesting that these measurements cannot differentiate LGITL and LPE. Similarly, although hypophosphatemia previously has been observed in cats with LPE,^39^ this finding was uncommon in cats in our study, being seen in only 2 (10%) LGITL cats and in no LPE cats. Future studies would be required to clarify the role of serum phosphorous concentration in the diagnosis of gastrointestinal tract disease in cats.

We also assessed a range of ultrasonographic variables, many of which did not differ between cats with LGITL and those with LPE. However, a range of altered ultrasonographic features suggesting abnormalities of the jejunal lymph node (eg, increased lymph node thickness, hypoechogenicity, rounded shape and hyperechoic perinodal fat) were more commonly seen in cats with LGITL than in cats with LPE. Although ultrasonographic features of jejunal lymphadenopathy previously have been associated with both LGITL and LPE,^26^ nodal ultrasonographic criteria that can discriminate LGITL from LPE have not been identified previously. Of all jejunal lymph node variables assessed, only rounded shape remained in the final multiple logistic regression model. However, this feature is somewhat subjective, and further work would be required to confirm its clinical relevance. Therefore, from a pragmatic point of view, it would be sensible for ultrasonographers to look for all features suggesting jejunal lymph node abnormality and not only rounded lymph node shape. One final feature of note was the presence of (albeit small volume) abdominal effusion, which was again more often seen in cats with LGITL. To our knowledge, abdominal effusion has not been reported previously as a variable that might help discriminate LGITL from LPE. Again, further work should be considered to confirm the clinical importance of this finding.

By simple statistical analyses, jejunal mucosal thickness was significantly higher in cats with LGITL (median, 1.4 mm) compared to LPE cats (median, 1.0 mm), although these variables were not included in the final multiple regression analyses. Previous studies also have identified thickening of the gastrointestinal wall and altered wall layering as some of the most common ultrasonographic findings in cats with both LGITL and LPE.^24^, ^26^ An increase in thickness of the muscularis layer relative to the other layers also was reported previously as the most common wall layering alteration in cats with LGITL,^26^ and also is commonly observed in cats with LPE and eosinophilic enteritis,^24^, ^47^ and occasionally is seen with mechanical obstruction.^27^ Although previous work has associated this finding with a diagnosis of LGITL rather than LPE, particularly in old cats,^26^ later work showed similar ultrasonographic wall layering differences in these 2 groups.^24^ Similar to a previous study,^24^ the jejunal muscularis layer was similar between groups (median, 1.1 mm) in our study. Therefore, it seems that not only can the muscularis layer thickening noted on ultrasound discriminate LGITL from LPE cats, but its origin also remains unknown based on current histological data.^31^ Further work is warranted to better characterize its origin.

The most infiltrated intestinal segment was the jejunum, both in cats diagnosed with LGITL (65% of biopsy samples with mucosal lymphoid infiltration came from the jejunum) and with LPE (88% of biopsy samples with mucosal leukocytic infiltration came from the jejunum; data presented in companion article).^31^ The primarily jejuno‐ileal involvement of lesions in our study was consistent with data published in other studies, where 86% to 100% of lesions were jejunal,^7^, ^12^ and 93% of lesions were ileal or at the ileocolic junction.^7^, ^12^ However, this segmental localization of intestinal lesions should be interpreted with caution because it might be associated with a preference in sampling site and, because biopsy samples were preferentially obtained by celiotomy, the number of sites sampled was limited. Therefore, the full extent of the lesions might not have been evident. This factor also might explain why clinical signs did not appear to correlate with the location of lesions. For example, many cats with jejunal lesions, ileal lesions or both did not have small intestinal diarrhea and instead were referred with signs of vomiting, anorexia, or weight loss.

Our study has several limitations. First, the number of cats included was small and, consequently, the study might have been underpowered for some variables. Second, although cats were prospectively included, the acquired ultrasonographic images were analyzed retrospectively after study completion. This approach enabled the analysis to be conducted in a blinded fashion, but it meant that such findings could not be integrated with other clinical findings as would usually happen. In addition, retrospective evaluation relied solely on available still images, which made the assessment of some criteria (eg, lesion distribution, presence, or absence of abdominal effusion) sometimes challenging. Furthermore, maximal severity of the lesions may have been underestimated occasionally. Third, it would have been useful for a control group of healthy cats to be included as a further comparator. As a result, additional studies are warranted to confirm our findings. Another limitation is that 3 cats (13%) had endoscopic biopsies, because the owners declined surgical sampling. As a result, jejunal neoplastic lesions may have been missed. A final limitation related the creation of an appropriate multiple regression model for clinical variables, not least because of confounding within the model, when hematochezia and polyphagia both were included. This situation is likely to have arisen because none of the 7 cats with hematochezia had polyphagia, and vice versa. The effect was that calculated ORs for the variables in the multiple regression model were unreliable, with some changing markedly from simple regression to multiple regression (eg, hematochezia OR of 0.127 in simple regression and OR < .001 in multiple regression). As a result, an alternative model was constructed after removal of hematochezia. Given this confounding, it is unclear as to whether a true difference exists in the presence of hematochezia between LGITL and LPE cats. A prospective study with a larger study population would help to clarify this uncertainty.

In conclusion, most clinical signs and usual laboratory results of cats diagnosed with LGITL or LPE overlap. However, male sex, duration of clinical signs, and polyphagia might help clinicians distinguish LGITL from LPE. Moreover, altered ultrasonographic features of the jejunal lymph node, particularly rounded shape, and the presence of (albeit small volume) abdominal effusion were more common in cats with LGITL compared to LPE cats. These findings could increase a clinician's index of suspicion for LGITL, enabling further investigations and treatment to be better tailored. For now, the final diagnosis still is based on thorough histopathology and immunohistochemistry assessment.

## CONFLICT OF INTEREST DECLARATION

Alexander J. German is an employee of the University of Liverpool, but his post is financially supported by Royal Canin, which is owned by Mars Petcare. Alexander J. German has also received financial remuneration for providing educational material, speaking at conferences, and consultancy work for Mars Petcare; all such remuneration has been for projects unrelated to the work reported in this manuscript. No other authors have a conflict of interest.

## OFF‐LABEL ANTIMICROBIAL USE DECLARATION

Authors declare no off‐label use of antimicrobials.

## INSTITUTIONAL ANIMAL CARE AND USE COMMITTEE (IACUC) OR OTHER APPROVAL DECLARATION

This study was approved by Ecole Nationale Vétérinaire d'Alfort's Ethical Committee (ENVA COMERC n°2017‐05‐09).

## HUMAN ETHICS APPROVAL DECLARATION

Authors declare human ethics approval was not needed for this study.

## Supporting information


**Table S1** Reference intervals for blood parameters analyzed by Idexx laboratories, France, and BioPôle laboratory at Alfort School of Veterinary Medicine, Paris, France.

## References

[1] Rissetto K , Villamil JA , Selting KA , Tyler J , Henry CJ . Recent trends in feline intestinal neoplasia: an epidemiologic study of 1,129 cases in the veterinary medical database from 1964 to 2004. J Am Anim Hosp Assoc. 2011;47:28‐36. 21164164 10.5326/JAAHA-MS-5554

[2] Ettinger SN . Principles of treatment for feline lymphoma. Clin Tech Small Anim Pract. 2003;18:98‐102. 12831069 10.1053/svms.2003.36623

[3] Gabor LJ , Malik R , Canfield PJ . Clinical and anatomical features of lymphosarcoma in 118 cats. Aust Vet J. 1998;76:725‐732. 9862061 10.1111/j.1751-0813.1998.tb12300.x

[4] Couto CG . What is new on feline lymphoma? J Feline Med Surg. 2001;3:171‐176. 11795953 10.1053/jfms.2001.0146 PMC10822295

[5] Barrs V , Beatty J . Feline alimentary lymphoma: 1. Classification, risk factors, clinical signs and non‐invasive diagnostics. J Feline Med Surg. 2012;14:182‐190. 22370860 10.1177/1098612X12439265 PMC10822432

[6] Moore PF , Rodriguez‐Bertos A , Kass PH . Feline gastrointestinal lymphoma: mucosal architecture, immunophenotype, and molecular clonality. Vet Pathol. 2012;49:658‐668. 21505197 10.1177/0300985811404712

[7] Paulin MV , Couronné L , Beguin J , et al. Feline low‐grade alimentary lymphoma: an emerging entity and a potential animal model for human disease. BMC Vet Res. 2018;14:306. 30305106 10.1186/s12917-018-1635-5 PMC6180644

[8] Freiche V. Novel extensive characterisation of feline low‐grade T‐cell intestinal lymphoma (T‐LGIL). In: Proceeding of the ECVIM 2019 Congress September 2019‐21. Milan, Italy; 2019.

[9] Chino J , Fujino Y , Kobayashi T , et al. Cytomorphological and immunological classification of feline lymphomas: clinicopathological features of 76 cases. J Vet Med Sci. 2013;75:701‐707. 23337319 10.1292/jvms.12-0246

[10] Russell KJ , Beatty JA , Dhand N , et al. Feline low‐grade alimentary lymphoma: how common is it? J Feline Med Surg. 2012;14:910‐912. 22811481 10.1177/1098612X12454861 PMC11108004

[11] Jergens AE . Feline idiopathic inflammatory bowel disease: what we know and what remains to be unraveled. J Feline Med Surg. 2012;14:445‐458. 22736679 10.1177/1098612X12451548 PMC10822384

[12] Lingard AE , Briscoe K , Beatty JA , et al. Low‐grade alimentary lymphoma: clinicopathological findings and response to treatment in 17 cases. J Feline Med Surg. 2009;11:692‐700. 19576832 10.1016/j.jfms.2009.05.021 PMC11132580

[13] Kiselow MA , Rassnick KM , McDonough SP , et al. Outcome of cats with low‐grade lymphocytic lymphoma: 41 cases (1995‐2005). J Am Vet Med Assoc. 2008;232:405‐410. 18241108 10.2460/javma.232.3.405

[14] Barrs V , Beatty J . Feline alimentary lymphoma. J Feline Med Surg. 2012;14:191‐201. 22370861 10.1177/1098612X12439266 PMC10822435

[15] Willard MD . Alimentary neoplasia in geriatric dogs and cats. Vet Clin N Am Small Anim Pract. 2012;42:693‐706‐vi. 10.1016/j.cvsm.2012.04.006 22720809

[16] Kiupel M , Smedley RC , Pfent C , et al. Diagnostic algorithm to differentiate lymphoma from inflammation in feline small intestinal biopsy samples. Vet Pathol. 2011;48:212‐222. 21149848 10.1177/0300985810389479

[17] Freiche V . Feline T‐cell low‐grade intestinal lymphoma: a novel model of lymphomagenesis according to the one‐health concept. In: 2020 ACVIM Forum on Demand Research Abstract Program. J Vet Intern Med. 2020;34:2830.

[18] Moore PF , Woo JC , Vernau W , Kosten S , Graham PS . Characterization of feline T cell receptor gamma (TCRG) variable region genes for the molecular diagnosis of feline intestinal T cell lymphoma. Vet Immunol Immunop. 2005;106:167‐178. 10.1016/j.vetimm.2005.02.014 15963816

[19] Briscoe KA , Krockenberger M , Beatty JA , et al. Histopathological and immunohistochemical evaluation of 53 cases of feline lymphoplasmacytic enteritis and low‐grade alimentary lymphoma. J Comp Pathol. 2011;145:187‐198. 21333999 10.1016/j.jcpa.2010.12.011

[20] Carreras JK , Goldschmidt M , Lamb M , McLear R , Drobatz KJ , Sørenmo KU . Feline epitheliotropic intestinal malignant lymphoma: 10 cases (1997‐2000). J Vet Intern Med. 2003;17:326‐331. 12774974 10.1111/j.1939-1676.2003.tb02456.x

[21] Mahony OM , Moore AS , Cotter SM , Engler SJ , Brown D , Penninck DG . Alimentary lymphoma in cats: 28 cases (1988‐1993). J Am Vet Med Assoc. 1995;207:1593‐1598. 7493898

[22] Waly NE , Gruffydd‐Jones TJ , Stokes CR , et al. Immunohistochemical diagnosis of alimentary lymphomas and severe intestinal inflammation in cats. J Comp Pathol. 2005;133:253‐260. 16213517 10.1016/j.jcpa.2005.05.004

[23] Castro‐López J , Teles M , Fierro C , Allenspach K , Planellas M , Pastor J . Pilot study: duodenal MDR1 and COX2 gene expression in cats with inflammatory bowel disease and low‐grade alimentary lymphoma. J Feline Med Surg. 2017;20:759‐766. 28948903 10.1177/1098612X17730708 PMC11104148

[24] Daniaux LA , Laurenson MP , Marks SL , et al. Ultrasonographic thickening of the muscularis propria in feline small intestinal small cell T‐cell lymphoma and inflammatory bowel disease. J Feline Med Surg. 2014;16:89‐98. 23900499 10.1177/1098612X13498596 PMC4177905

[25] Evans SE , Bonczynski JJ , Broussard JD , Han E , Baer KE . Comparison of endoscopic and full‐thickness biopsy specimens for diagnosis of inflammatory bowel disease and alimentary tract lymphoma in cats. J Am Vet Med Assoc. 2006;229:1447‐1450. 17078807 10.2460/javma.229.9.1447

[26] Zwingenberger AL , Marks SL , Baker TW , Moore PF . Ultrasonographic evaluation of the muscularis propria in cats with diffuse small intestinal lymphoma or inflammatory bowel disease. J Vet Intern Med. 2010;24:289‐292. 20102493 10.1111/j.1939-1676.2009.0457.x

[27] Diana A , Pietra M , Guglielmini C , Boari A , Bettini G , Cipone M . Ultrasonographic and pathologic features of intestinal smooth muscle hypertrophy in four cats. Vet Radiol Ultrasound. 2003;44:566‐569. 14599170 10.1111/j.1740-8261.2003.tb00508.x PMC7169284

[28] Donato PD , Penninck D , Pietra M , et al. Ultrasonographic measurement of the relative thickness of intestinal wall layers in clinically healthy cats. J Feline Med Surg. 2014;16:333‐339. 24174500 10.1177/1098612X13509080 PMC11383109

[29] Hahn H , Freiche V , Baril A , et al. Ultrasonographic, endoscopic and histological appearance of the caecum in clinically healthy cats. J Feline Med Surg. 2017;19:85‐93. 26316516 10.1177/1098612X15602740 PMC10816570

[30] Freiche V , Cordonnier N , Paulin MV , et al. Feline low‐grade intestinal T cell lymphoma: a unique natural model of human indolent T cell lymphoproliferative disorder of the gastrointestinal tract. Lab Invest. 2021;101(6):794‐804. 33692440 10.1038/s41374-021-00581-x

[31] Freiche V , Paulin MV , Cordonnier N , et al. Histopathologic, phenotypic, and molecular criteria to discriminate low‐grade intestinal T‐cell lymphoma in cats from lymphoplasmacytic enteritis in cats. J Vet Intern Med. Forthcoming. 2021. 10.1111/jvim.16231 PMC8692189 34374109

[32] Nyman HT , O'Brien RT . The sonographic evaluation of lymph nodes. Clin Tech Small Anim Pract. 2007;22(3):128‐137. 17844819 10.1053/j.ctsap.2007.05.007

[33] Washabau RJ , Day MJ , Willard MD , et al. Endoscopic, biopsy, and histopathologic guidelines for the evaluation of gastrointestinal inflammation in companion animals. J Vet Intern Med. 2010;24:10‐26. 20391635 10.1111/j.1939-1676.2009.0443.x

[34] Swerdlow SH , Campo E , Pileri SA , et al. The 2016 revision of the World Health Organization classification of lymphoid neoplasms. Blood. 2016;127:2375‐2390. 26980727 10.1182/blood-2016-01-643569 PMC4874220

[35] Uzal FA , Plattner BL , Hastetter JM . Alimentary system. Jubb, Kennedy & Palmer's Pathology of Domestic Animals. Vol 2. Philadelphia, United States: Elsevier; 2015:1‐257.

[36] Raftery AE . Bayesian model selection in social research. Am Sociol Assoc. 1995;25:111‐163.

[37] Zwahlen CH , Lucroy MD , Kraegel SA , Madewell BR . Results of chemotherapy for cats with alimentary malignant lymphoma: 21 cases (1993‐1997). J Am Vet Med Assoc. 1998;213:1144‐1149. 9787382

[38] Wilson HM . Feline alimentary lymphoma: demystifying the enigma. Top Companion Anim Med. 2008;23:177‐184. 19081551 10.1053/j.tcam.2008.10.003

[39] Jergens AE , Crandell JM , Evans R , Ackermann M , Miles KG , Wang C . A clinical index for disease activity in cats with chronic enteropathy. J Vet Intern Med. 2010;24:1027‐1033. 20584141 10.1111/j.1939-1676.2010.0549.x

[40] Castro‐López J , Ramis A , Planellas M , Teles M , Pastor J . Cyclooxygenase‐2 immunoexpression in intestinal epithelium and lamina propria of cats with inflammatory bowel disease and low‐grade alimentary lymphoma. BMC Vet Res. 2018;14:158. 29764431 10.1186/s12917-018-1486-0 PMC5952374

[41] French RA , Seitz SE , Valli VE . Primary epitheliotropic alimentary T‐cell lymphoma with hepatic involvement in a dog. Vet Pathol. 1996;33:349‐352. 8740712 10.1177/030098589603300315

[42] Chott A , Dragosics B , Radaszkiewicz T . Peripheral T‐cell lymphomas of the intestine. Am J Pathol. 1992;141:1361‐1371. 1466400 PMC1886751

[43] Dieter RS , Duque K . Enterotherapy associated T‐cell lymphoma: a case report and literature review. WMJ Official Publ State Med Soc Wis. 2000;99:28‐31. 11089447

[44] Gieger T . Alimentary lymphoma in cats and dogs. Vet Clin N Am Small Anim Pract. 2011;41:419‐432. 10.1016/j.cvsm.2011.02.001 21486644

[45] Richter KP . Feline gastrointestinal lymphoma. Vet Clin N Am Small Anim Pract. 2003;33:1083‐1098. 10.1016/s0195-5616(03)00054-8 14552162

[46] Fondacaro JV , Richter KP , Carpenter JL , et al. Feline gastrointestinal lymphomas: 67 cases (1988‐1996). Eur J Comp Gastroenterol. 1999;4(2):5‐11.

[47] Tucker S , Penninck DG , Keating JH , Webster CRL . Clinicopathological and ultrasonographic features of cats with eosinophilic enteritis. J Feline Med Surg. 2014;16:950‐956. 24591305 10.1177/1098612X14525385 PMC11104094

