# Supplementary material for: Clinical, laboratory and ultrasonographic findings differentiating low‐grade intestinal T‐cell lymphoma from lymphoplasmacytic enteritis in cats
Source: J Vet Intern Med. 2021 Oct 23;35(6):2685–96. doi: 10.1111/jvim.16272 (PMC8692195; doi:10.1111/jvim.16272)
Supplement: Supplementary file 1 — Table S1 Reference intervals for blood parameters analyzed by Idexx laboratories, France, and BioPôle laboratory at Alfort School of Veterinary Medicine, Paris, France. [file JVIM-35-2685-s001.pdf]

Supplementary Table 1: Reference intervals for blood parameters analyzed by Idexx® laboratories, France, and BioPôle laboratory at Alfort School of Veterinary Medicine, Paris, France.

Blood parameters	Reference intervals	
	**BioPôle laboratory**	**Idexx®**
Alanine aminotransferase (ALT) (U/L)	15 - 123	< 175
Alkaline phosphatase (ALP) (U/L)	22 - 187	< 73
Total protein (g/dL)	6.4 – 9.6	5.9 – 8.7
Albumin (g/dL)	2.9 – 3.5	2.7 – 4.4
Feline pancreatic lipase immuno-reactivity (f-PL) (µg/L)	/	≤ 3,5
Cobalamin (Vitamin B12) (ng/L)	/	200 – 1.680[1]
Inorganic phosphorus (mmol/L)	1.03 – 2.51	0.8 – 2.2
Hemoglobin (mmol/L)	6.08 – 10.49	5.59 – 9.31
Leucocytes (G/L)	3.7 - 19	5 - 19
Lymphocytes (G/L)	1.1 – 10.36	1 - 6
Neutrophils (G/L)	1.45 – 9.62	3 - 11
Eosinophils (G/L)	0.16 – 1.9	0.09 – 2.18
Monocytes (G/L)	0.09 – 0.82	0.04 – 0.53

[1]Hypocobalaminemia was defined by a serum cobalamin concentration < 200 ng/L, since was the detection limit of the assay.
